# Impact of Neoadjuvant Chemotherapy on Prognosis of Patients with Mucinous Gastric Adenocarcinoma: A Propensity Score Matching Study

**DOI:** 10.1245/s10434-025-19069-9

**Published:** 2026-04-27

**Authors:** Yiming Lu, Danni Zhu, Ke Shen, Jiahui Chen, Xiangdong Cheng

**Affiliations:** 1 The Second Clinical Medical College of Zhejiang, Chinese Medical University, Hangzhou, China; 2 Department of Gastric Surgery, Zhejiang Cancer Hospital, Hangzhou, China; 3 Wenzhou Medical University, Wenzhou, China

**Keywords:** Mucinous gastric adenocarcinoma, Gastric adenocarcinoma, Neoadjuvant chemotherapy, Overall survival, Tumor regression grade

## Abstract

**Background:**

Mucinous gastric adenocarcinoma (MGC) is a rare, aggressive malignancy. This study aimed to explore whether MGC is associated with poorer survival compared with nonmucinous gastric adenocarcinoma (NMGC) and to further investigate the potential impact of neoadjuvant chemotherapy (NAC) on MGC by assessing overall survival (OS), tumor regression grade (TRG), and histological subtypes.

**Methods:**

A retrospective analysis was conducted on 492 patients with MGC and 7945 patients with NMGC undergoing resection at Zhejiang Cancer Hospital (2014–2023). Patients were categorized into neoadjuvant and surgery groups. MGC was further classified as pure (≥ 80%) or mixed (< 80%). OS was analyzed using Kaplan–Meier and Cox regression, with propensity score matching (PSM) for adjustment.

**Results:**

After PSM, OS did not differ between MGC and NMGC. Patients with MGC receiving NAC had significantly worse OS than those undergoing surgery, with NAC as an independent risk factor. TRG analysis showed a markedly lower proportion of responders (TRG 0–1) in MGC than NMGC, indicating reduced chemosensitivity. Among subtypes, NAC remained an adverse factor in pure MGC, while mixed MGC showed no significant difference after PSM.

**Conclusions:**

MGC exhibits poor pathological response and no survival benefit from NAC, supporting upfront surgery as the preferred approach. TRG findings highlight intrinsic chemoresistance, particularly in pure MGC, underscoring the need for histology-based individualized strategies.

**Supplementary Information:**

The online version contains supplementary material available at 10.1245/s10434-025-19069-9.

Gastric cancer (GC) is a globally prevalent malignant tumor, with an annual incidence of approximately 980,000 new cases and 660,000 associated deaths.^1^ According to the Lauren classification, gastric cancer is categorized into intestinal type and diffuse type.^2^ Intestinal-type gastric cancer has a higher incidence and a relatively favorable prognosis. In contrast, diffuse-type gastric cancer is characterized by diffuse growth, loss of cell junctions, lack of glandular formation, and poor differentiation. Mucinous gastric adenocarcinoma (MGC),^3^ a distinct histological subtype, is classified under diffuse-type gastric cancer and accounts for 2.6_6.6% of all gastric cancer cases.^4^

MGC is generally regarded as highly invasive, exhibiting features such as low early detection rates, deep infiltration, high lymph node metastasis rates, and frequent peritoneal dissemination.^5,6^ However, the prognostic differences between MGC and nonmucinous gastric adenocarcinoma (NMGC) remain controversial, with some studies reporting worse outcomes for MGC,^4,7^ while others have found no significant survival difference.^7–9^ The majority of existing studies on MGC prognosis have focused on the influence of various clinicopathological characteristics,^10,11^ while fewer investigations have explored the prognostic differences between surgery following neoadjuvant chemotherapy (NAC) and surgery first.

In other types of mucinous carcinomas, such as rectal mucinous carcinoma, studies have demonstrated no significant difference in the 5-year survival rates between neoadjuvant chemoradiotherapy and surgery first.^12,13^ In esophageal mucinous carcinoma, NAC has been associated with enhanced survival outcomes.^14^ However, the impact of NAC on the prognosis of MGC remains uncertain, necessitating this investigation into the survival changes among patients with MGC following NAC.

This study aims to examine differences in overall survival (OS) between patients with MGC and patients with NMGC, between patients with MGC who received NAC and those who underwent surgery first, as well as between pure MGC and mixed MGC. The results are anticipated to provide preliminary insights into the role of NAC in MGC prognosis and contribute to clinical evidence for optimizing treatment strategies for patients with MGC, with a focus on individualized approaches based on tumor regression grade (TRG) and histological subtype.

## Methods

### Patient Selection

#### Ethical Approval

**Fig. 1:**
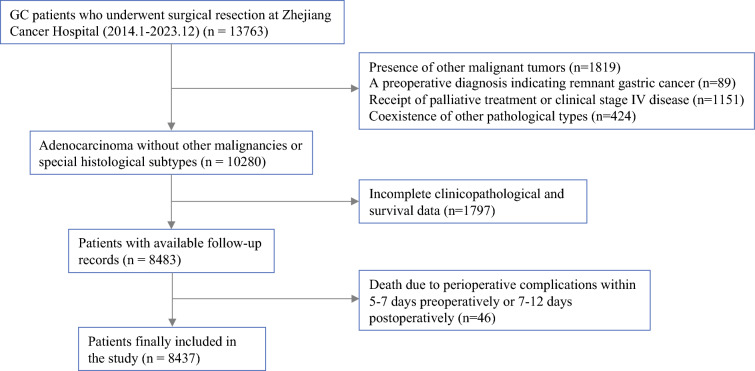
Patient selection and exclusion process

This study was approved by the Ethics Committee of Zhejiang Cancer Hospital (IRB-2025-554 [IIT]). We conducted a retrospective analysis of data from patients with MGC who underwent surgical resection at Zhejiang Cancer Hospital between January 2014 and December 2023. Patients were included if they met the following criteria: (1) pathologically confirmed pure MGC or tumors containing MGC components, (2) underwent curative-intent or palliative gastrectomy, and (3) had complete clinicopathological and survival data available. Patients were excluded if they met any of the following criteria: (1) presence of other malignant tumors, (2) a preoperative diagnosis indicating remnant gastric cancer, (3) receipt of palliative treatment or clinical stage Ⅳ disease, (4) coexistence of other pathological types (e.g., signet-ring cell carcinoma, neuroendocrine carcinoma), or (5) death due to perioperative complications within 5–7 days preoperatively or 7–12 days postoperatively (Fig. 1).

Patients with NMGC undergoing surgical resection were randomly selected. Patients were included if they had pathologically confirmed gastric adenocarcinoma without MGC components. Furthermore, all other inclusion and exclusion criteria were consistent with those applied to patients with MGC.

### Follow-Up

Patients underwent follow-up evaluations at least twice annually. For patients unable to attend routine postoperative evaluations at our center, survival status was monitored via telephone follow-ups.

### Histopathological Analysis

Pathologists specialized in gastrointestinal oncology evaluated all surgical specimens. According to the World Health Organization (WHO) classification, MGC is defined as an adenocarcinoma containing abundant extracellular mucin that accounts for more than 50% of the tumor volume. However, this threshold may not fully reflect prognostic heterogeneity. Therefore, on the basis of the pathological classification proposed by Meng et al., an 80% cutoff was applied to distinguish pure MGC (≥ 80% mucinous component) from mixed MGC (< 80%).^15^

Tumor regression following NAC was evaluated using the TRG system as recommended by National Comprehensive Cancer Network (NCCN) guidelines. TRG 0 indicates complete response with no viable tumor cells (including lymph nodes), TRG 1 indicates near-complete response with only single cells or small clusters, TRG 2 indicates partial response with residual tumor and evident regression, and TRG 3 indicates poor or no response with extensive residual cancer. Patients with TRG 0–1 were considered good responders, whereas those with TRG 2–3 were classified as poor responders.

### Definition of Variables and Groups

In this study, patients were categorized into either the surgical group or the neoadjuvant group depending on whether they had undergone NAC prior to surgery. Additionally, patients with MGC were divided into two subgroups: pure MGC and mixed MGC, on the basis of histological features. According to the TRG system, patients who received NAC were further stratified into two subgroups: TRG 0–1 (good responders) and TRG 2–3 (poor responders). Clinical tumor–node–metastasis (cTNM) stage was utilized to assess patients in both groups. Overall survival (OS) was defined as the interval from the date of diagnosis to the last follow-up, the time of death, or the end of the follow-up period (e.g., loss to follow-up or death due to unrelated causes).

To address potential confounders, we performed propensity score matching (PSM) using various clinicopathological variables. The following variables were included in the PSM model: age, body mass index (BMI), sex, family history, smoking history, alcohol consumption, NAC, adjuvant chemotherapy, tumor size, lymphovascular invasion, perineural invasion, tumor location, histological differentiation, surgical procedure, clinical T stage, and clinical N stage. These variables were selected because they are well-established clinicopathological factors known to influence survival outcomes in gastric cancer and could potentially confound the relationship between treatment modality (NAC or upfront surgery) and overall survival. Including these variables allowed us to minimize baseline imbalances and ensure comparability between matched groups.

### Statistical Analysis

PSM was conducted using R version 4.2. A logistic regression model was employed to estimate propensity scores for group assignment, with a caliper width of 0.2 of the standard deviation of the logit of the propensity score. The 1:1 nearest-neighbor matching method was subsequently employed to achieve balanced cohort matching. All statistical analyses were conducted using SPSS version 24.0 (IBM, USA). Categorical variables and continuous variables were compared using the chi-squared test and the Mann–Whitney *U* test, respectively. Survival curves were estimated using the Kaplan–Meier method, and differences in survival between groups were assessed using the log-rank test. A multivariate Cox proportional hazards model was applied to identify prognostic factors for survival outcomes. A two-tailed *P*-value of less than 0.05 was considered statistically significant.

## Results

### Patient Selection

Between January 2014 and December 2023, a total of 492 patients with MGC and 7945 patients with NMGC who underwent surgical resection at Zhejiang Cancer Hospital were included in this study.

Among the 492 patients with MGC who met the inclusion criteria, 65 received NAC, while the remaining 427 received surgery directly. On the basis of histopathological classification, MGC was further divided into pure mucinous gastric adenocarcinoma (pure MGC, ≥ 80% MGC) and mixed mucinous gastric adenocarcinoma (mixed MGC, < 80% MGC). In the neoadjuvant group, there were 20 cases of pure MGC and 45 cases of mixed MGC, while in the surgery group, there were 68 cases of pure MGC and 359 cases of mixed MGC. Additionally, we randomly selected 7945 patients with gastric adenocarcinoma who underwent surgical resection. Among them, 1068 patients received NAC, whereas 6877 were treated with surgery directly. According to the NCCN TRG system, patients with MGC and patients with NMGC in the neoadjuvant group were further categorized into TRG 0–1 (good responders) and TRG 2–3 (poor responders). Specifically, there were 4 patients with MGC and 312 patients with NMGC in the TRG 0–1 subgroup, and 61 patients with MGC and 756 patients with NMGC in the TRG 2–3 subgroup.

### PSM Results

#### Comparison of OS Between Patients with MGC and Patients with NMGC

**Fig. 2:**
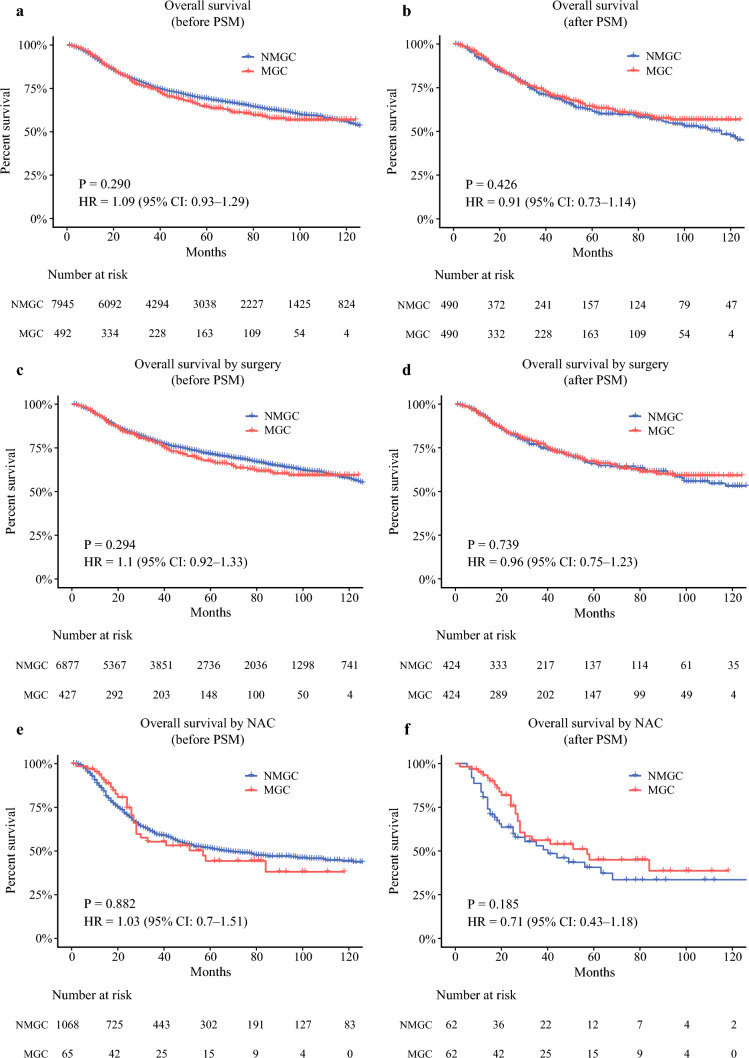
Comparison of OS between patients with MGC and patients with NMGC, with subgroup analyses in the surgery and neoadjuvant groups. **a** OS comparison between patients with MGC and patients with NMGC before PSM. **b** OS comparison between patients with MGC and patients with NMGC after PSM. **c** OS comparison between patients with MGC and patients with NMGC in the surgery group before PSM. **d** OS comparison between patients with MGC and patients with NMGC in the surgery group after PSM. **e** OS comparison between patients with MGC and patients with NMGC in the neoadjuvant group before PSM. **f** OS comparison between patients with MGC and patients with NMGC in the neoadjuvant group after PSM. MGC, mucinous gastric adenocarcinoma; NMGC, nonmucinous gastric adenocarcinoma; PSM, propensity score matching; NAC, neoadjuvant chemotherapy

To assess whether OS differs between patients with MGC and patients with NMGC, we compared the survival curves of the two groups using the Kaplan–Meier method (Fig. 2). Before PSM, the comparison of OS between patients with MGC and patients with NMGC showed no statistically significant difference (Fig. 2a, P = 0.290), indicating that, in the unmatched cohort, the OS outcomes of the two groups were comparable.

We first performed a comparative analysis of baseline clinicopathological characteristics between the two groups. The results showed no significant differences in BMI, sex, smoking history, alcohol consumption, NAC, surgical procedure, or tumor location, suggesting that patients with MGC and patients with NMGC were comparable in these aspects (Table 1). However, several features differed significantly between the groups. Specifically, compared with patients with NMGC, those with MGC were older, had a lower proportion of positive family history, a higher proportion receiving adjuvant chemotherapy, and larger tumor size. In addition, patients with MGC exhibited higher proportions of lymphovascular invasion, perineural invasion, and poorly differentiated tumors, all with statistically significant differences. Furthermore, the two groups also showed significant differences in tumor invasion depth, regional lymph node involvement, and overall TNM stage, indicating that patients with MGC tended to present with more advanced disease-related features, including larger tumor burden and more aggressive pathological characteristics.

**Table 1:** Comparison of clinicopathological characteristics before and after PSM of patients with MGC

Patient characteristic	Before PSM			After PSM		
	NMGC (*n* = 7945)	MGC (*n* = 492)	*P* value	NMGC (*n* = 490)	MGC (*n* = 490)	*P* value
Age (years), mean ± SD	62.58 ± 10.02	64.03 ± 8.93	<0.001	64.30 ± 9.97	63.99 ± 8.92	0.610
BMI (kg/m^2^), mean ± SD	22.20 ± 3.02	22.22 ± 3.04	0.912	22.19 ± 2.95	22.22 ± 3.05	0.901
Sex, *n* (%)			0.956			0.706
Female	1898 (23.89)	117 (23.78)		112 (22.86)	117 (23.88)	
Male	6047 (76.11)	375 (76.22)		378 (77.14)	373 (76.12)	
Family history, *n* (%)			< 0.001			0.233
No	4822 (60.69)	357 (72.56)		338 (68.98)	355 (72.45)	
Yes	3123 (39.31)	135 (27.44)		152 (31.02)	135 (27.55)	
Smoking history, *n* (%)			0.780			0.654
No	4379 (55.12)	268 (54.47)		260 (53.06)	267 (54.49)	
Yes	3566 (44.88)	224 (45.53)		230 (46.94)	223 (45.51)	
Alcohol consumption, *n* (%)			0.800			0.302
No	5301 (66.72)	331 (67.28)		344 (70.20)	329 (67.14)	
Yes	2644 (33.28)	161 (32.72)		146 (29.80)	161 (32.86)	
NAC, *n* (%)			0.884			0.852
No	6877 (86.56)	427 (86.79)		423 (86.33)	425 (86.73)	
Yes	1068 (13.44)	65 (13.21)		67 (13.67)	65 (13.27)	
Adjuvant chemotherapy, *n* (%)			< 0.001			0.839
No	4095 (51.54)	167 (33.94)		164 (33.47)	167 (34.08)	
Yes	3850 (48.46)	325 (66.06)		326 (66.53)	323 (65.92)	
Tumor size, *n* (%)			< 0.001			0.250
<5 cm	5170 (65.07)	250 (50.81)		232 (47.35)	250 (51.02)	
≥ 5 cm	2775 (34.93)	242 (49.19)		258 (52.65)	240 (48.98)	
Lymphovascular invasion, *n* (%)			< 0.001			0.302
No	4442 (55.91)	219 (44.51)		203 (41.43)	219 (44.69)	
Yes	3503 (44.09)	273 (55.49)		287 (58.57)	271 (55.31)	
Perineural invasion, *n* (%)			0.025			0.565
No	4433 (55.80)	249 (50.61)		239 (48.78)	248 (50.61)	
Yes	3512 (44.20)	243 (49.39)		251 (51.22)	242 (49.39)	
Tumor location, *n* (%)			0.532			0.437
Upper third	1792 (22.56)	113 (22.97)		112 (22.86)	112 (22.86)	
Middle third	1874 (23.59)	104 (21.14)		89 (18.16)	104 (21.22)	
Lower third	4187 (52.70)	271 (55.08)		281 (57.35)	270 (55.10)	
Entire	92 (1.16)	4 (0.81)		8 (1.63)	4 (0.82)	
Differentiation, *n* (%)			< 0.001			0.627
Low	4370 (55.00)	379 (77.03)		370 (75.51)	377 (76.94)	
Moderate	3308 (41.64)	100 (20.33)		110 (22.45)	100 (20.41)	
Well	267 (3.36)	13 (2.64)		10 (2.04)	13 (2.65)	
Surgical procedure, *n* (%)			0.240			0.563
Open	5583 (70.27)	358 (72.76)		364 (74.29)	356 (72.65)	
Laparoscopic	2362 (29.73)	134 (27.24)		126 (25.71)	134 (27.35)	
cT, *n* (%)			< 0.001			0.898
T1	1844 (23.21)	52 (10.57)		47 (9.59)	52 (10.61)	
T2	1119 (14.08)	70 (14.23)		77 (15.71)	70 (14.29)	
T3	1195 (15.04)	119 (24.19)		116 (23.67)	117 (23.88)	
T4	3787 (47.67)	251 (51.02)		250 (51.02)	251 (51.22)	
cN, *n* (%)			< 0.001			0.776
N0	2960 (37.26)	110 (22.36)		104 (21.22)	110 (22.45)	
N1	1522 (19.16)	141 (28.66)		138 (28.16)	139 (28.37)	
N2	1839 (23.15)	134 (27.24)		148 (30.20)	134 (27.35)	
N3	1624 (20.44)	107 (21.75)		100 (20.41)	107 (21.84)	
cTNM stage, *n* (%)			< 0.001			0.567
I	2168 (27.29)	67 (13.62)		58 (11.84)	67 (13.67)	
II	1689 (21.26)	98 (19.92)		108 (22.04)	98 (20.00)	
III	4088 (51.45)	327 (66.46)		324 (66.12)	325 (66.33)	

*BMI* body mass index, *MGC* mucinous gastric adenocarcinoma, *NMGC* nonmucinous gastric adenocarcinoma, *PSM* propensity score matching

To address these baseline imbalances, we performed PSM to balance key clinicopathological factors between the two groups. Kaplan–Meier survival analysis of the matched cohorts again revealed no significant difference in OS between patients with MGC and patients with NMGC (Fig. 2b, P = 0.426), suggesting that, even after adjusting for baseline characteristics, the two histological subtypes did not differ significantly in OS.

#### Comparison of OS Between Patients with MGC and Patients with NMGC in the Surgery and Neoadjuvant Groups

We divided all patients into a surgery group and a neoadjuvant group, and analyzed the survival curves of patients with MGC and patients with NMGC within each group (Fig. 2). In the surgery group, the OS of patients with MGC was not significantly different from that of patients with NMGC (Fig. 2c, P = 0.294). Similarly, in the neoadjuvant group, there was also no significant difference in OS between patients with MGC and patients with NMGC (Fig. 2e, P = 0.882).

In terms of clinicopathological baseline characteristics, patients with MGC in the surgery group were older, had a lower proportion of family history, higher proportions of adjuvant chemotherapy, larger tumor size, vascular invasion, lymph node metastasis, advanced gastric cancer, poorer differentiation, and deeper tumor infiltration compared with patients with NMGC in the surgery group. In the neoadjuvant group, patients with MGC had larger tumor size, higher proportions of vascular invasion, and poorer differentiation than patients with NMGC. PSM was performed again to minimize differences in baseline characteristics between the two groups. Detailed clinical information after matching is shown in Supplementary Tables S1 and S2.

After PSM, no significant difference in OS was observed between patients with MGC and patients with NMGC in the surgery group (Fig. 2d, P = 0.739). Similarly, the OS of patients with MGC and patients with NMGC in the neoadjuvant group did not differ significantly (Fig. 2f, P = 0.185).

#### Comparison of OS between Patients with MGC in the Neoadjuvant Group and Those in the Surgery Group

**Fig. 3:**
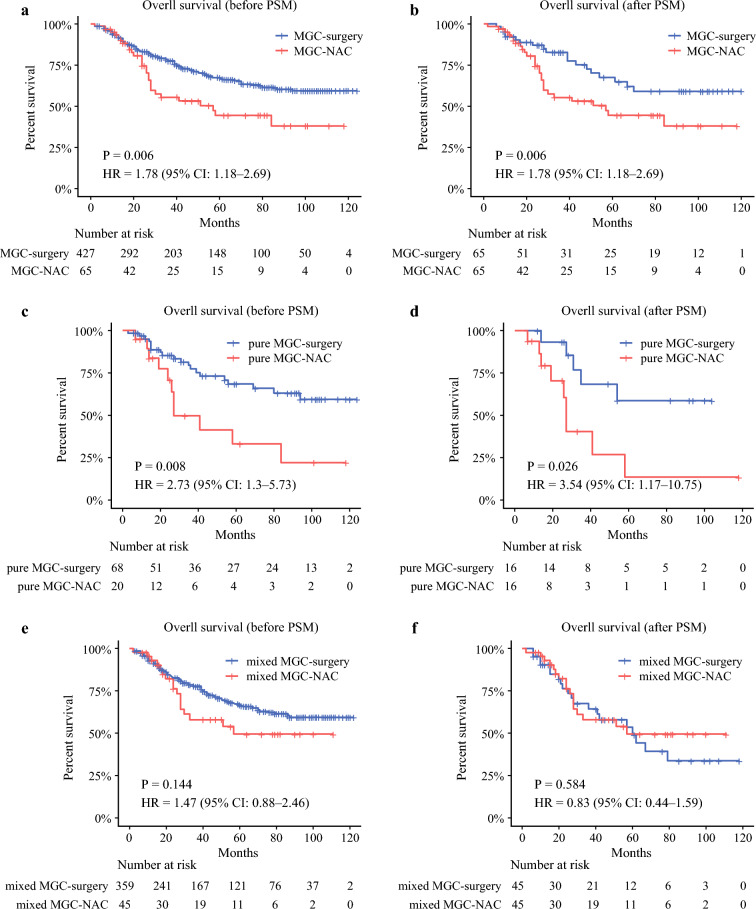
Comparison of OS between patients with MGC in the surgery and neoadjuvant groups, with subgroup analyses in the pure and mixed groups. **a** OS comparison between patients with MGC in the surgery and neoadjuvant groups before PSM. **b** OS comparison between patients with MGC in the surgery and neoadjuvant groups after PSM. **c** OS comparison between patients with pure MGC in the surgery and neoadjuvant groups before PSM. **d** OS comparison between patients with pure MGC in the surgery and neoadjuvant groups after PSM. **e** OS comparison between patients with mixed MGC in the surgery and neoadjuvant groups before PSM. **f** OS comparison between patients with mixed MGC in the surgery and neoadjuvant groups after PSM. MGC, mucinous gastric adenocarcinoma; NMGC, nonmucinous gastric adenocarcinoma; PSM, propensity score matching; NAC, neoadjuvant chemotherapy

To evaluate the impact of NAC on OS in patients with MGC, the cohort was stratified into a neoadjuvant group (*n* = 65) and a surgery group (*n* = 427), and their survival curves were analyzed (Fig. 3). The results revealed a statistically significant difference, with poorer survival observed in the neoadjuvant group (Fig. 3a, P = 0.006). In terms of baseline clinicopathological characteristics, patients in the neoadjuvant group had a higher proportion of open surgery and postoperative adjuvant chemotherapy, as well as more advanced clinical stage compared with those in the surgery group.

PSM was subsequently conducted to ensure baseline comparability, matching 65 patients in the neoadjuvant group with 65 in the surgery group (Table 2). The results showed that OS in the neoadjuvant group remained significantly poorer than that in the surgery group (Fig. 3b, P = 0.035). Multivariate Cox regression analysis performed for all patients with MGC identified NAC and perineural invasion as independent prognostic factors, with NAC emerging as an independent risk factor. Patients in the neoadjuvant group had a significantly increased risk of death compared with those in the surgery group (hazard ratio (HR) = 1.99, 95% confidence interval (CI) 1.09–3.60, *P* = 0.024; Table 3). Subgroup analysis was then conducted to compare OS between patients with pure MGC and patients with mixed MGC. The OS of patients with pure MGC in the neoadjuvant group was significantly worse than that of those in the surgery group (Fig. 3c, P = 0.008). In contrast, no significant difference in OS was observed between the mixed MGC neoadjuvant and surgery groups (Fig. 3e, P = 0.144). Regarding baseline clinicopathological characteristics, patients with pure MGC in the neoadjuvant group showed more advanced clinical staging than those in the surgery group. For patients with mixed MGC, those in the neoadjuvant group had a higher proportion of open surgery and postoperative adjuvant chemotherapy, as well as more advanced clinical stage compared with the surgery group. Detailed clinicopathological characteristics before and after PSM are presented in Supplementary Tables S3 and S4.

**Table 2:** Comparison of clinicopathological characteristics before and after PSM of patients with MGC

Patient characteristic	Before PSM			After PSM		
	Surgical (*n* = 427)	Neoadjuvant (*n* = 65)	*P* value	Surgical (*n* = 65)	Neoadjuvant (*n* = 65)	*P* value
Age (years), mean ± SD	64.08 ± 9.07	63.71 ± 8.02	0.755	63.20 ± 9.36	63.71 ± 8.02	0.740
BMI (kg/m^2^), mean ± SD	22.29 ± 3.10	21.76 ± 2.61	0.187	21.96 ± 3.07	21.76 ± 2.61	0.682
Sex, *n* (%)			0.427			0.260
Female	99 (23.19)	18 (27.69)		24 (36.92)	18 (27.69)	
Male	328 (76.81)	47 (72.31)		41 (63.08)	47 (72.31)	
Family history, *n* (%)			0.584			0.837
No	308 (72.13)	49 (75.38)		50 (76.92)	49 (75.38)	
Yes	119 (27.87)	16 (24.62)		15 (23.08)	16 (24.62)	
Smoking history, *n* (%)			0.219			0.357
No	228 (53.40)	40 (61.54)		45 (69.23)	40 (61.54)	
Yes	199 (46.60)	25 (38.46)		20 (30.77)	25 (38.46)	
Alcohol consumption, *n* (%)			0.939			0.070
No	287 (67.21)	44 (67.69)		53 (81.54)	44 (67.69)	
Yes	140 (32.79)	21 (32.31)		12 (18.46)	21 (32.31)	
Adjuvant chemotherapy, *n* (%)			< 0.001			1.000
No	158 (37.00)	9 (13.85)		9 (13.85)	9 (13.85)	
Yes	269 (63.00)	56 (86.15)		56 (86.15)	56 (86.15)	
Tumor size, *n* (%)			0.181			0.380
<5 cm	222 (51.99)	28 (43.08)		33 (50.77)	28 (43.08)	
≥ 5 cm	205 (48.01)	37 (56.92)		32 (49.23)	37 (56.92)	
Lymphovascular invasion, *n* (%)			0.803			0.380
No	191 (44.73)	28 (43.08)		33 (50.77)	28 (43.08)	
Yes	236 (55.27)	37 (56.92)		32 (49.23)	37 (56.92)	
Perineural invasion, *n* (%)			0.441			0.479
No	219 (51.29)	30 (46.15)		26 (40.00)	30 (46.15)	
Yes	208 (48.71)	35 (53.85)		39 (60.00)	35 (53.85)	
Tumor location, *n* (%)			0.628			0.516
Upper third	97 (22.72)	16 (24.62)		20 (30.77)	16 (24.62)	
Middle third	87 (20.37)	17 (26.15)		12 (18.46)	17 (26.15)	
Lower third	239 (55.97)	32 (49.23)		33 (50.77)	32 (49.23)	
Entire	4 (0.94)	0 (0.00)				
Differentiation,* n* (%)			0.356			0.349
Low	327 (76.58)	52 (80.00)		56 (86.15)	52 (80.00)	
Moderate	87 (20.37)	13 (20.00)		9 (13.85)	13 (20.00)	
Well	13 (3.04)	0 (0.00)				
Surgical procedure, *n* (%)			0.001			0.545
Open	300 (70.26)	58 (89.23)		60 (92.31)	58 (89.23)	
Laparoscopic	127 (29.74)	7 (10.77)		5 (7.69)	7 (10.77)	
cTNM stage, *n* (%)			< 0.001			0.784
I	67 (15.69)	0 (0.00)		0 (0.00)	0 (0.00)	
II	91 (21.31)	7 (10.77)		8 (12.31)	7 (10.77)	
III	269 (63.00)	58 (89.23)		57 (87.69)	58 (89.23)	

*BMI* body mass index, *MGC* mucinous gastric adenocarcinoma, *PSM* propensity score matching

**Table 3:** Univariate and multivariate analyses of overall survival after PSM in patients with MGC

Patient characteristic	Univariate analysis			Multivariate analysis		
	HR	95% CI	*P* value	HR	95% CI	*P* value
Age (years), mean	3.63	0.48–27.48	0.211			
BMI (kg/m^2^), mean	0.89	0.79–1.00	0.055			
Sex						
Female	1.00	1.00	–			
Male	1.36	0.73–2.54	0.338			
Family history						
No	1.00	1.00	–			
Yes	0.93	0.47–1.84	0.839			
Smoking history						
No	1.00	1.00	–			
Yes	1.11	0.61–2.02	0.727			
Alcohol consumption						
No	1.00	1.00	–			
Yes	0.84	0.42–1.70	0.632			
NAC						
No	1.00	1.00	–	1.00	1.00	–
Yes	1.88	1.05–3.37	0.035	1.99	1.09-3.60	0.024
Adjuvant chemotherapy						
No	1.00	1.00	–	1.00	1.00	
Yes	0.44	0.22–0.89	0.023	0.53	0.26–1.08	0.080
Tumor size						
<5 cm	1.00	1.00	–			–
≥ 5 cm	1.15	0.65–2.03	0.643			
Lymphovascular invasion						
No	1.00	1.00	–			–
Yes	1.39	0.78–2.48	0.262			
Perineural invasion						
No	1.00	1.00	–	1.00	1.00	–
Yes	1.99	1.09–3.64	0.025	2.11	1.13–3.91	0.018
Tumor location						
Upper third	1.00	1.00	–			–
Middle third	1.14	0.50–2.59	0.749			
Lower third	1.08	0.54–2.17	0.818			
Differentiation						
Low	1.00	1.00	–			–
Moderate	0.41	0.16–1.05	0.062			
Surgical procedure						
Open	1.00	1.00	–			
Laparoscopic	1.30	0.40–4.23	0.665			
cTNM stage						
II	1.00	1.00	–			–
III	2.58	0.80–8.35	0.113			

*BMI* body mass index, *MGC* mucinous gastric adenocarcinoma, *CI* confidence interval, *HR* hazard ratio

To minimize potential confounding, PSM was performed to balance baseline characteristics between groups. After matching, OS in the pure MGC neoadjuvant group remained significantly worse than that in the pure MGC surgery group (Fig. 3d, P = 0.026), while no significant difference in OS was observed between the mixed MGC neoadjuvant group and the mixed MGC surgery group (Fig. 3f, P = 0.584). Further multivariate analysis restricted to patients with pure MGC confirmed that NAC was an independent risk factor for poor prognosis compared with surgery first (HR = 3.36, 95% CI 1.10–10.24, *P* = 0.033; Table 4).

**Table 4:** Univariate and multivariate analyses of overall survival after PSM in patients with pure MGC

Patient characteristic	Univariate analysis			Multivariate analysis		
	HR	95% CI	*P* value	HR	95% CI	*P* value
Age (years), mean	1.03	0.96–1.11	0.340			
BMI (kg/m^2^), mean	0.91	0.74–1.13	0.407			
Sex						
Female	1.00	1.00	–			
Male	0.90	0.25–3.28	0.875			
Family history						
No	1.00	1.00	–			
Yes	1.45	0.40–5.24	0.566			
Smoking history						
No	1.00	1.00	–			
Yes	1.82	0.62–5.33	0.276			
Alcohol consumption						
No	1.00	1.00	–			
Yes	0.88	0.24–3.16	0.839			
NAC						
No	1.00	1.00	–	1.00	1.00	–
Yes	3.54	1.17–10.75	0.026	3.36	1.10–10.24	0.033
Adjuvant chemotherapy						
No	1.00	1.00	–	1.00	1.00	
Yes	0.08	0.01–0.60	0.014	0.09	0.01–0.66	0.018
Tumor size						
<5 cm	1.00	1.00	–			
≥ 5 cm	0.75	0.23–2.42	0.630			
Lymphovascular invasion						
No	1.00	1.00	–			
Yes	1.33	0.46–3.85	0.602			
Perineural invasion						
No	1.00	1.00	–			
Yes	1.19	0.41–3.40	0.751			
Tumor location						
Upper third	1.00	1.00	–			
Middle third	1.57	0.26–9.52	0.625			
Lower third	1.68	0.36–7.85	0.507			
Differentiation						
Low	1.00	1.00	–			
Moderate	0.90	0.20–4.02	0.886			
Surgical procedure						
Open	1.00	1.00	–			
Laparoscopic	2.10	0.46–9.62	0.340			
cTNM stage						
II	1.00	1.00	–			
III	4.52	0.59–580.97	0.185			

*BMI* body mass index, *MGC* mucinous gastric adenocarcinoma, *CI* confidence interval, *HR* hazard ratio

#### TRG-Related Analysis in the Neoadjuvant Group

**Fig. 4:**
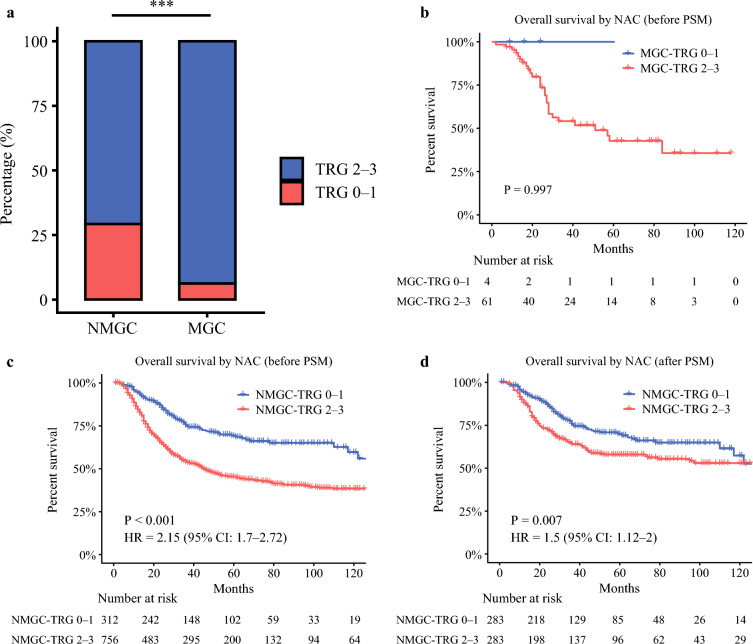
Comparison of TRG between patients with MGC and patients with NMGC in the neoadjuvant group, and survival analysis within TRG subgroups. **a** Tumor regression grade (TRG) distribution in patients with MGC and patients with NMGC in the neoadjuvant group. **b** OS comparison between patients with MGC with TRG 0–1 and TRG 2–3 in the neoadjuvant group before PSM. **c** OS comparison between patients with NMGC with TRG 0–1 and TRG 2–3 in the neoadjuvant group before PSM. **d** OS comparison between patients with NMGC with TRG 0–1 and TRG 2–3 in the neoadjuvant group after PSM

To investigate the pathological response to NAC, we compared the distribution of TRG scores between patients with MGC and patients with NMGC. The chi-squared test revealed a significant difference in the proportion of TRG categories between the two groups (Fig. 4a, P < 0.001). These results indicate that patients with MGC exhibited a significantly poorer pathological response to NAC compared with patients with NMGC.

To further assess the prognostic implications of TRG, OS was compared between TRG subgroups within the neoadjuvant group. In patients with MGC, only four cases achieved TRG 0–1, and all remained alive at the last follow-up; therefore, survival comparison between TRG 0–1 and TRG 2–3 subgroups was not feasible (Fig. 4b, P = 0.997). In contrast, patients with NMGC demonstrated a significant difference in OS between TRG 0–1 and TRG 2–3 subgroups before PSM (Fig. 4c, P < 0.001). After matching for baseline characteristics, the survival difference remained significant (Fig. 4d, P = 0.007). These findings suggest that TRG is a strong prognostic indicator of survival in patients with NMGC following NAC, whereas its prognostic value in MGC may be limited owing to the low rate of good pathological response.

## Discussion

MGC, as a distinct subtype of gastric cancer, is typically associated with a higher degree of malignancy. It is characterized by a low early diagnosis rate, large tumor size, deep invasion depth, high lymph node metastasis rate, and a high frequency of peritoneal dissemination.^5,6^ These features present significant challenges in clinical management. In our study, the OS of patients with MGC was comparable to that of patients with NMGC before PSM. After adjusting for baseline factors using PSM, no significant prognostic difference was observed between the two groups. The prognosis of MGC remains controversial. Some studies suggest that MGC has a worse prognosis than NMGC, particularly in the absence of effective treatment, leading to lower survival rates. However, mucinous histology itself has not been identified as an independent prognostic factor for gastric cancer.^4,10^ Additionally, some studies indicate that MGC is often diagnosed at a more advanced stage compared with NMGC, contributing to its poorer prognosis. When matched for stage, no significant difference in prognosis between patients with MGC and patients with NMGC has been observed.^7^ Ahn et al. conducted a multivariate analysis and concluded that tumor stage was the only independent risk factor for recurrence-free survival (RFS) and OS. Subgroup analysis based on tumor stage revealed no significant differences in RFS or OS between the MGC and NMGC groups at any stage. These findings suggest that the lower RFS and OS observed in patients with MGC may be attributed to the more advanced stage at initial diagnosis rather than the mucinous histology itself.^8^ Similarly, Komori et al., after performing PSM for clinicopathological characteristics, found no significant difference in the 5-year OS and cancer-specific survival (CSS) between patients with MGC and patients with NMGC.^9^ These findings are consistent with our results after adjusting for tumor stage. Furthermore, we stratified patients with MGC and patients with NMGC into surgical and neoadjuvant subgroups for comparison, and no significant differences in OS were observed.

We further investigated the potential efficacy of NAC in MGC by comparing the prognostic impact of NAC followed by surgery versus surgery first. Currently, studies on NAC for mucinous adenocarcinoma have primarily focused on the colon, rectum, and appendix, with limited reports on gastric mucinous adenocarcinoma. Caspers et al. analyzed patients enrolled in the D1/D2 and CRITICS clinical trials, categorizing them into surgery cohort and NAC cohort. They compared the histopathological response and survival rates of mucinous gastric cancer (muc-GC) with those of intestinal-type gastric cancer (int-GC) and diffuse-type gastric cancer (dif-GC) across these cohorts. Their findings revealed that, in the surgery cohort, the 5-year survival rate of muc-GC was slightly better than that of dif-GC but worse than that of int-GC. In the NAC cohort, muc-GC demonstrated greater tumor regression compared with both int-GC and dif-GC. Notably, both muc-GC and int-GC were associated with significantly better survival outcomes than dif-GC. These findings suggest that muc-GC responds more favorably to NAC and has a more favorable prognosis relative to dif-GC.^16^ However, this study did not directly compare the surgery-only and NAC cohorts. Additionally, potential downstaging effects of chemotherapy were not accounted for in the surgery-only group, and no clinical or pathological adjustments were made for baseline disparities between the three cohorts, which may have affected result accuracy. In our study, we directly compared patients with MGC undergoing surgery first with those receiving NAC. The survival curves between the two groups showed a significant difference, with the neoadjuvant group exhibiting worse prognosis. After using PSM to balance clinicopathological differences, a significant survival difference between the two groups remained, consistent with the pre-PSM results. Further multivariate regression analysis identified NAC and perineural invasion as independent prognostic factors, with NAC emerging as an independent risk factor. The hazard ratio (HR) for the neoadjuvant group was 1.99 times higher than that for the surgery group. To our knowledge, this is the first study to report the impact of NAC versus surgery first on the survival of patients with MGC. Our findings indicate that NAC does not prolong OS in patients with MGC; instead, those in the surgery-only group had longer survival times.

To further clarify the pathological response to NAC, we compared the TRG distribution between MGC and NMGC. The results revealed a markedly poorer pathological response in MGC (TRG 0–1: 6.2%) compared with NMGC (TRG 0–1: 29.2%), suggesting that MGC is substantially less sensitive to chemotherapy. Survival analysis showed that patients with NMGC with TRG 0–1 achieved significantly better OS than those with TRG 2–3, both before and after PSM. However, owing to the extremely small number of patients with MGC with TRG 0–1—all alive at last follow-up—the prognostic role of TRG in MGC could not be definitively assessed. These findings indicate that the poor chemotherapy responsiveness of MGC, reflected by a predominance of high TRG grades, may partly explain the lack of NAC benefit in this population.

NAC has been a major research focus in improving cancer prognosis. In general, this approach has been widely recognized for its beneficial effects in various malignancies. However, not all tumor types respond favorably to NAC, as certain cancers may exhibit resistance or reduced sensitivity to treatment. Therefore, further exploration of the efficacy and influencing factors of NAC across different tumor types is crucial for optimizing personalized treatment strategies and improving patient survival.

Current research suggests that the development of chemoresistance in MGC may involve a combination of Mucin 2 (MUC2)-mediated mechanisms and alterations in the tumor microenvironment. MUC2, a secreted mucin that is frequently overexpressed in MGC compared with NMGC, has been reported to act as a physical barrier limiting the penetration of chemotherapeutic agents such as 5-fluorouracil (5-FU).^17–23^ Its highly glycosylated structure facilitates the formation of a hydrated layer and steric hindrance, which further reduces drug diffusion and bioavailability, while high MUC2 expression has also been associated with resistance to apoptosis and decreased sensitivity to chemotherapy in various epithelial cancers.^24,25^ Experimental studies have demonstrated that attenuation of MUC2 expression significantly increased apoptosis in mucinous colon cancer models,^26^ suggesting a functional role in chemoresistance. Genomic data further indicate that MUC2 overexpression often coexists with TP53 mutations, observed in approximately 41% of MGC cases, which may impair DNA repair capacity and increase tolerance to cytotoxic agents. Co-expression with genes such as GPR120 (FFAR4) and B3GNT6 also implies the potential involvement of fatty acid metabolism and glycosylation in drug resistance. Beyond tumor-intrinsic mechanisms, the tumor microenvironment contributes importantly: in microsatellite stable (MSS) MGC, cancer-associated fibroblasts (CAFs) are enriched and secrete cytokines such as interleukin (IL)-6 and transforming growth factor (TGF)-β, which support tumor survival and may attenuate chemotherapy efficacy. From an immunological perspective, about 19% of MGCs exhibit a microsatellite instability-high (MSI-H) phenotype. While MSI-H tumors often respond well to immune checkpoint inhibitors, they are typically less sensitive to conventional chemotherapy agents such as 5-FU and oxaliplatin.^17^ Moreover, the mucin barrier may restrict immune cell infiltration, including T cells and natural killer cells, thereby creating a “cold tumor” phenotype that further limits the immunomodulatory effects of chemotherapy.^27,28^

Additionally, the toxicity induced by chemotherapy can significantly impair a patient’s physical condition and contribute to secondary immunodeficiency. In clinical settings, such adverse effects are often offset by substantial survival benefits.^29^ However, when chemotherapy fails to improve survival outcomes, its toxic impact may exert a dual detrimental effect: suppressing antitumor immune responses and altering the tumor microenvironment in ways that confer a selective growth advantage to cancer cells. This vicious cycle may accelerate disease progression, leading to more aggressive tumor behavior, shortened progression-free survival, and ultimately increased all-cause mortality.^30^ This may help explain why patients with MGC undergoing NAC exhibit poorer OS compared with those treated with surgery first.

In the subtype analysis of MGC, we found that patients with pure MGC in the neoadjuvant group had significantly worse OS than those in the surgery group (*P* = 0.026). Further multivariate regression analysis focusing on patients with pure MGC identified NAC as the only independent prognostic risk factor. Specifically, the risk of death in patients receiving NAC was 3.36 times higher than that in those undergoing surgery first. In contrast, after baseline matching, there was no significant difference in OS between the neoadjuvant and surgery-only groups in patients with mixed MGC (*P* = 0.584). This discrepancy may be attributed to biological differences between MGCs with varying mucin content.

In colorectal mucinous adenocarcinoma, both pure and mixed types show high expression of goblet cell markers such as MUC2, but pure-type tumors exhibit more uniform mucinous differentiation, whereas mixed types are more heterogeneous and biologically complex.^31,32^ Similar heterogeneity is also observed in MGC. Meng et al. reported that pure MGC demonstrated fewer vascular and lymphatic invasions and better survival than mixed-type tumors.^15^

In our study, we found that patients with a higher proportion of mucinous components tended to have a poorer pathological response to NAC. In contrast, mixed MGC showed no significant survival difference between the NAC and surgery groups. These findings suggest that the degree of mucinous differentiation may be inversely correlated with chemosensitivity in gastric cancer.

Therefore, further molecular and omics-based investigations are needed to elucidate the mechanisms underlying this reduced chemotherapy response in highly mucinous tumors. A comprehensive understanding of the biological basis of MGC will facilitate the identification of potential therapeutic targets and contribute to the development of individualized treatment strategies.

Naturally, this study has several limitations. First, as a single-center retrospective analysis, the sample size is relatively small, especially for the MGC TRG 0–1 subgroup, which limits the statistical power of our analysis. Although efforts were made to include as many eligible patients as possible, the limited number of cases may restrict the generalizability of our findings. Despite the use of PSM to minimize confounding, selection bias cannot be entirely excluded. Additionally, TRG assessment, while standardized by NCCN criteria, remains somewhat subjective and observer-dependent, which may introduce variability in classifying pathological responses. Future prospective, multicenter studies with larger cohorts and more standardized pathological assessments are warranted to validate the role of NAC in patients with MGC. Moreover, owing to the lack of recurrence data and detailed chemotherapy regimen information, we were unable to evaluate progression-free survival or compare the efficacy of different chemotherapy protocols. Given the observed heterogeneity in survival outcomes—particularly between pure and mixed MGC subtypes and their differing responses to NAC—comprehensive clinical endpoints, including recurrence patterns, treatment response, and molecular characteristics, should be integrated into future studies. This will facilitate a more complete understanding of treatment impact and allow for the optimization of individualized therapeutic strategies for MGC.

## Conclusions

Patients with MGC derive no survival benefit from NAC, exhibiting poorer OS than those receiving upfront surgery. The markedly lower proportion of TRG 0–1 in MGC compared with NMGC underscores its intrinsic chemoresistance, suggesting that standard NAC regimens may be less effective for this histological subtype. Upfront surgery may therefore represent a more suitable strategy for resectable MGC. Furthermore, among MGC subtypes, NAC remained an independent adverse factor in pure MGC, whereas mixed MGC showed only a limited survival difference after adjustment. These findings emphasize the need for histology-based, individualized treatment approaches in gastric cancer.

## Supplementary Information

Below is the link to the electronic supplementary material.

Supplementary file1 (DOCX 59 kb)
