# Supplementary material for: Impact of Neoadjuvant Chemotherapy on Prognosis of Patients with Mucinous Gastric Adenocarcinoma: A Propensity Score Matching Study
Source: Ann Surg Oncol. 2026 Apr 27;33(7):6572–86. doi: 10.1245/s10434-025-19069-9 (PMC13242480; doi:10.1245/s10434-025-19069-9)
Supplement: Supplementary file 1 — Supplementary file1 (DOCX 59 kb) [file 10434_2025_19069_MOESM1_ESM.docx]

**Supplement Table 1** Comparison of clinicopathological characteristics before and after PSM of MGC patients in surgical group

| Patient Characteristics | Before PSM | | |  | After PSM | | |  | |
| --- | --- | --- | --- | --- | --- | --- | --- | --- | --- |
|  | NMGC  (n=6877) | MGC  (n=427) | *P* value |  | NMGC  (n=424) | MGC  (n=424) | *P* value |  | |
| Age, Mean ± SD | 62.85 ± 10.04 | 64.08 ± 9.07 | 0.013 |  | 63.87 ± 10.01 | 64.09 ± 9.08 | 0.741 |  | |
| BMI, Mean ± SD | 22.26 ± 3.03 | 22.29 ± 3.10 | 0.816 |  | 22.15 ± 3.12 | 22.27 ± 3.10 | 0.576 |  | |
| Sex, n (%) |  |  | 0.593 |  |  |  | 0.809 |  | |
| Female | 1673 (24.33) | 99 (23.19) |  |  | 102 (24.06) | 99 (23.35) |  |  | |
| Male | 5204 (75.67) | 328 (76.81) |  |  | 322 (75.94) | 325 (76.65) |  |  | |
| Family history, n (%) |  |  | <0.001 |  |  |  | 0.939 |  | |
| No | 4163 (60.54) | 308 (72.13) |  |  | 304 (71.70) | 305 (71.93) |  |  | |
| Yes | 2714 (39.46) | 119 (27.87) |  |  | 120 (28.30) | 119 (28.07) |  |  | |
| Smoking history, n (%) |  |  | 0.467 |  |  |  | 0.582 |  | |
| No | 3796 (55.20) | 228 (53.40) |  |  | 220 (51.89) | 228 (53.77) |  |  | |
| Yes | 3081 (44.80) | 199 (46.60) |  |  | 204 (48.11) | 196 (46.23) |  |  | |
| Alcohol consumption, n (%) |  |  | 0.818 |  |  |  | 0.771 |  | |
| No | 4585 (66.67) | 287 (67.21) |  |  | 281 (66.27) | 285 (67.22) |  |  | |
| Yes | 2292 (33.33) | 140 (32.79) |  |  | 143 (33.73) | 139 (32.78) |  |  | |
| Adjuvant chemotherapy, n (%) |  |  | <0.001 |  |  |  | 0.887 |  | |
| No | 3891 (56.58) | 158 (37.00) |  |  | 156 (36.79) | 158 (37.26) |  |  | |
| Yes | 2986 (43.42) | 269 (63.00) |  |  | 268 (63.21) | 266 (62.74) |  |  | |
| Tumor size, n (%) |  |  | <0.001 |  |  |  | 0.837 |  | |
| ＜5cm | 4544 (66.08) | 222 (51.99) |  |  | 219 (51.65) | 222 (52.36) |  |  | |
| ≥5cm | 2333 (33.92) | 205 (48.01) |  |  | 205 (48.35) | 202 (47.64) |  |  | |
| Lymphovascular invasion, n (%) |  |  | <0.001 |  |  |  | 0.945 |  | |
| No | 3746 (54.47) | 191 (44.73) |  |  | 189 (44.58) | 190 (44.81) |  |  | |
| Yes | 3131 (45.53) | 236 (55.27) |  |  | 235 (55.42) | 234 (55.19) |  |  | |
| Perineural invasion, n (%) |  |  | 0.101 |  |  |  | 0.891 |  | |
| No | 3807 (55.36) | 219 (51.29) |  |  | 219 (51.65) | 217 (51.18) |  |  | |
| Yes | 3070 (44.64) | 208 (48.71) |  |  | 205 (48.35) | 207 (48.82) |  |  | |
| Tumor location, n (%) |  |  | 0.535 |  |  |  | 0.614 |  | |
| Upper third | 1496 (21.75) | 97 (22.72) |  |  | 94 (22.17) | 95 (22.41) |  |  | |
| Middle third | 1615 (23.48) | 87 (20.37) |  |  | 76 (17.92) | 87 (20.52) |  |  | |
| Lower third | 3701 (53.82) | 239 (55.97) |  |  | 252 (59.43) | 238 (56.13) |  |  | |
| Entire | 65 (0.95) | 4 (0.94) |  |  | 2 (0.47) | 4 (0.94) |  |  | |
| Differentiation, n (%) |  |  | <0.001 |  |  |  | 0.658 |  | |
| Low | 3701 (53.82) | 327 (76.58) |  |  | 320 (75.47) | 324 (76.42) |  |  | |
| Moderate | 2927 (42.56) | 87 (20.37) |  |  | 86 (20.28) | 87 (20.52) |  |  | |
| Well | 249 (3.62) | 13 (3.04) |  |  | 18 (4.25) | 13 (3.07) |  |  | |
| Surgical procedure, n (%) |  |  | 0.378 |  |  |  | 0.650 |  | |
| Open | 4691 (68.21) | 300 (70.26) |  |  | 304 (71.70) | 298 (70.28) |  |  | |
| Laparoscopic | 2186 (31.79) | 127 (29.74) |  |  | 120 (28.30) | 126 (29.72) |  |  | |
| cT, n (%) |  |  | <0.001 |  |  |  | 0.997 |  | |
| T1 | 1750 (25.45) | 52 (12.18) |  |  | 50 (11.79) | 52 (12.26) |  |  | |
| T2 | 1035 (15.05) | 66 (15.46) |  |  | 66 (15.57) | 66 (15.57) |  |  | |
| T3 | 998 (14.51) | 113 (26.46) |  |  | 110 (25.94) | 110 (25.94) |  |  | |
| T4 | 3094 (44.99) | 196 (45.90) |  |  | 198 (46.70) | 196 (46.23) |  |  | |
| cN, n (%) |  |  | <0.001 |  |  |  | 0.901 |  | |
| N0 | 2745 (39.92) | 107 (25.06) |  |  | 101 (23.82) | 107 (25.24) |  |  | |
| N1 | 1333 (19.38) | 130 (30.44) |  |  | 124 (29.25) | 127 (29.95) |  |  | |
| N2 | 1478 (21.49) | 105 (24.59) |  |  | 114 (26.89) | 105 (24.76) |  |  | |
| N3 | 1321 (19.21) | 85 (19.91) |  |  | 85 (20.05) | 85 (20.05) |  |  | |
| cTNM stage, n (%) |  |  | <0.001 |  |  |  | 0.990 |  | |
| I | 2064 (30.01) | 67 (15.69) |  |  | 66 (15.57) | 67 (15.80) |  |  | |
| II | 1508 (21.93) | 91 (21.31) |  |  | 90 (21.23) | 91 (21.46) |  |  | |
| III | 3305 (48.06) | 269 (63.00) |  |  | 268 (63.21) | 266 (62.74) |  |  | |
| **BMI**: Body Mass Index; **MGC**: mucinous gastric adenocarcinoma; **NMGC**: non-mucinous gastric adenocarcinoma; **PSM**: Propensity Score Matching | | | | | | | | |  |

**Supplement Table 2** Comparison of clinicopathological characteristics before and after PSM of MGC patients in neoadjuvant group

| Patient Characteristics | Before PSM | | |  | After PSM | | |  | |
| --- | --- | --- | --- | --- | --- | --- | --- | --- | --- |
|  | NMGC  (n=1068) | MGC  (n=65) | *P* value |  | NMGC  (n=62) | MGC  (n=62) | *P* value |  | |
| Age, Mean ± SD | 60.84 ± 9.71 | 63.71 ± 8.02 | 0.020 |  | 63.29 ± 8.34 | 63.35 ± 7.91 | 0.224 |  | |
| BMI, Mean ± SD | 21.88 ± 2.87 | 21.76 ± 2.61 | 0.740 |  | 21.96 ± 3.17 | 21.66 ± 2.63 | 0.513 |  | |
| Sex, n (%) |  |  | 0.206 |  |  |  | 0.551 |  | |
| Female | 225 (21.07) | 18 (27.69) |  |  | 14 (22.58) | 16 (25.81) |  |  | |
| Male | 843 (78.93) | 47 (72.31) |  |  | 48 (77.42) | 46 (74.19) |  |  | |
| Family history, n (%) |  |  | 0.027 |  |  |  | 0.430 |  | |
| No | 659 (61.70) | 49 (75.38) |  |  | 43 (69.35) | 46 (74.19) |  |  | |
| Yes | 409 (38.30) | 16 (24.62) |  |  | 19 (30.65) | 16 (25.81) |  |  | |
| Smoking history, n (%) |  |  | 0.274 |  |  |  | 0.856 |  | |
| No | 583 (54.59) | 40 (61.54) |  |  | 33 (53.23) | 37 (59.68) |  |  | |
| Yes | 485 (45.41) | 25 (38.46) |  |  | 29 (46.77) | 25 (40.32) |  |  | |
| Alcohol consumption, n (%) |  |  | 0.914 |  |  |  | 0.849 |  | |
| No | 716 (67.04) | 44 (67.69) |  |  | 45 (72.58) | 41 (66.13) |  |  | |
| Yes | 352 (32.96) | 21 (32.31) |  |  | 17 (27.42) | 21 (33.87) |  |  | |
| Adjuvant chemotherapy, n (%) |  |  | 0.292 |  |  |  | 0.473 |  | |
| No | 204 (19.10) | 9 (13.85) |  |  | 12 (19.35) | 9 (14.52) |  |  | |
| Yes | 864 (80.90) | 56 (86.15) |  |  | 50 (80.65) | 53 (85.48) |  |  | |
| Tumor size, n (%) |  |  | 0.014 |  |  |  | 0.364 |  | |
| ＜5cm | 626 (58.61) | 28 (43.08) |  |  | 22 (35.48) | 27 (43.55) |  |  | |
| ≥5cm | 442 (41.39) | 37 (56.92) |  |  | 40 (64.52) | 35 (56.45) |  |  | |
| Lymphovascular invasion, n (%) |  |  | <0.001 |  |  |  | 0.858 |  | |
| No | 696 (65.17) | 28 (43.08) |  |  | 22 (35.48) | 28 (45.16) |  |  | |
| Yes | 372 (34.83) | 37 (56.92) |  |  | 40 (64.52) | 34 (54.84) |  |  | |
| Perineural invasion, n (%) |  |  | 0.048 |  |  |  | 0.476 |  | |
| No | 626 (58.61) | 30 (46.15) |  |  | 30 (48.39) | 30 (48.39) |  |  | |
| Yes | 442 (41.39) | 35 (53.85) |  |  | 32 (51.61) | 32 (51.61) |  |  | |
| Tumor location, n (%) |  |  | 0.545 |  |  |  | 0.198 |  | |
| Upper third | 296 (27.72) | 16 (24.62) |  |  | 15 (24.19) | 16 (25.81) |  |  | |
| Middle third | 259 (24.25) | 17 (26.15) |  |  | 11 (17.74) | 16 (25.81) |  |  | |
| Lower third | 486 (45.51) | 32 (49.23) |  |  | 36 (58.06) | 30 (48.39) |  |  | |
| Entire | 27 (2.53) | 0 (0.00) |  |  | 0 (0.00) | 0 (0.00) |  |  | |
| Differentiation, n (%) |  |  | 0.016 |  |  |  | 0.823 |  | |
| Low | 669 (62.64) | 52 (80.00) |  |  | 50 (80.65) | 49 (79.03) |  |  | |
| Moderate | 381 (35.67) | 13 (20.00) |  |  | 12 (19.35) | 13 (20.97) |  |  | |
| Well | 18 (1.69) | 0 (0.00) |  |  | 0 (0.00) | 0 (0.00) |  |  | |
| Surgical procedure, n (%) |  |  | 0.225 |  |  |  | 1.000 |  | |
| Open | 892 (83.52) | 58 (89.23) |  |  | 55 (88.71) | 55 (88.71) |  |  | |
| Laparoscopic | 176 (16.48) | 7 (10.77) |  |  | 7 (11.29) | 7 (11.29) |  |  | |
| cT, n (%) |  |  | 0.006 |  |  |  | 0.886 |  | |
| T1 | 94 (8.80) | 0 (0.00) |  |  | 0 (0.00) | 0 (0.00) |  |  | |
| T2 | 84 (7.87) | 4 (6.15) |  |  | 5 (8.06) | 4 (6.45) |  |  | |
| T3 | 197 (18.45) | 6 (9.23) |  |  | 7 (11.29) | 6 (9.68) |  |  | |
| T4 | 693 (64.89) | 55 (84.62) |  |  | 50 (80.65) | 52 (83.87) |  |  | |
| cN, n (%) |  |  | 0.015 |  |  |  | 0.932 |  | |
| N0 | 215 (20.13) | 3 (4.62) |  |  | 3 (4.84) | 3 (4.84) |  |  | |
| N1 | 189 (17.70) | 11 (16.92) |  |  | 12 (19.35) | 11 (17.74) |  |  | |
| N2 | 361 (33.80) | 29 (44.62) |  |  | 23 (37.10) | 27 (43.55) |  |  | |
| N3 | 303 (28.37) | 22 (33.85) |  |  | 24 (38.71) | 21 (33.87) |  |  | |
| cTNM stage, n (%) |  |  | 0.007 |  |  |  | 0.783 |  | |
| I | 104 (9.74) | 0 (0.00) |  |  | 0 (0.00) | 0 (0.00) |  |  | |
| II | 181 (16.95) | 7 (10.77) |  |  | 8 (12.90) | 7 (11.29) |  |  | |
| III | 783 (73.31) | 58 (89.23) |  |  | 54 (87.10) | 55 (88.71) |  |  | |
| **BMI**: Body Mass Index; **MGC**: mucinous gastric adenocarcinoma; **NMGC**: non-mucinous gastric adenocarcinoma; **PSM**: Propensity Score Matching | | | | | | | | |  |

**Supplement Table 3** Comparison of clinicopathological characteristics before and after PSM of pure MGC patients

| Patient Characteristics | Before PSM | | |  | After PSM | | |  | |
| --- | --- | --- | --- | --- | --- | --- | --- | --- | --- |
|  | Surgical  (n=68) | Neoadjuvant  (n=20) | *P* value |  | Surgical  (n=31) | Neoadjuvant (n=16) | *P* value |  | |
| Age, Mean ± SD | 62.72 ± 9.48 | 62.60 ± 7.54 | 0.959 |  | 62.75 ± 11.28 | 62.88 ± 7.75 | 0.971 |  | |
| BMI, Mean ± SD | 21.93 ± 2.72 | 21.73 ± 2.75 | 0.772 |  | 22.43 ± 3.37 | 21.95 ± 2.40 | 0.646 |  | |
| Sex, n (%) |  |  | 0.815 |  |  |  | 1.000 |  | |
| Female | 14 (20.59) | 3 (15.00) |  |  | 2 (12.50) | 3 (18.75) |  |  | |
| Male | 54 (79.41) | 17 (85.00) |  |  | 14 (87.50) | 13 (81.25) |  |  | |
| Family history, n (%) |  |  | 0.682 |  |  |  | 1.000 |  | |
| No | 56 (82.35) | 15 (75.00) |  |  | 13 (81.25) | 13 (81.25) |  |  | |
| Yes | 12 (17.65) | 5 (25.00) |  |  | 3 (18.75) | 3 (18.75) |  |  | |
| Smoking history, n (%) |  |  | 0.728 |  |  |  | 1.000 |  | |
| No | 37 (54.41) | 10 (50.00) |  |  | 8 (50.00) | 9 (56.25) |  |  | |
| Yes | 31 (45.59) | 10 (50.00) |  |  | 8 (50.00) | 7 (43.75) |  |  | |
| Alcohol consumption, n (%) |  |  | 0.661 |  |  |  | 1.000 |  | |
| No | 44 (64.71) | 14 (70.00) |  |  | 12 (75.00) | 11 (68.75) |  |  | |
| Yes | 24 (35.29) | 6 (30.00) |  |  | 4 (25.00) | 5 (31.25) |  |  | |
| Adjuvant chemotherapy, n (%) |  |  | 0.214 |  |  |  | 1.000 |  | |
| No | 18 (26.47) | 2 (10.00) |  |  | 1 (6.25) | 2 (12.50) |  |  | |
| Yes | 50 (73.53) | 18 (90.00) |  |  | 15 (93.75) | 14 (87.50) |  |  | |
| Tumor size, n (%) |  |  | 0.259 |  |  |  | 0.433 |  | |
| ＜5cm | 30 (44.12) | 6 (30.00) |  |  | 3 (18.75) | 6 (37.50) |  |  | |
| ≥5cm | 38 (55.88) | 14 (70.00) |  |  | 13 (81.25) | 10 (62.50) |  |  | |
| Lymphovascular invasion, n (%) |  |  | 0.182 |  |  |  | 1.000 |  | |
| No | 42 (61.76) | 9 (45.00) |  |  | 7 (43.75) | 7 (43.75) |  |  | |
| Yes | 26 (38.24) | 11 (55.00) |  |  | 9 (56.25) | 9 (56.25) |  |  | |
| Perineural invasion, n (%) |  |  | 0.781 |  |  |  | 1.000 |  | |
| No | 35 (51.47) | 11 (55.00) |  |  | 8 (50.00) | 8 (50.00) |  |  | |
| Yes | 33 (48.53) | 9 (45.00) |  |  | 8 (50.00) | 8 (50.00) |  |  | |
| Tumor location, n (%) |  |  | 0.432 |  |  |  | 0.785 |  | |
| Upper third | 12 (17.65) | 2 (10.00) |  |  | 4 (25.00) | 2 (12.50) |  |  | |
| Middle third | 15 (22.06) | 7 (35.00) |  |  | 3 (18.75) | 4 (25.00) |  |  | |
| Lower third | 41 (60.29) | 11 (55.00) |  |  | 9 (56.25) | 10 (62.50) |  |  | |
| Differentiation, n (%) |  |  | 0.815 |  |  |  | 1.000 |  | |
| Low | 54 (79.41) | 17 (85.00) |  |  | 14 (87.50) | 13 (81.25) |  |  | |
| Moderate | 14 (20.59) | 3 (15.00) |  |  | 2 (12.50) | 3 (18.75) |  |  | |
| Surgical procedure, n (%) |  |  | 0.635 |  |  |  | 0.484 |  | |
| Open | 56 (82.35) | 18 (90.00) |  |  | 16 (100.00) | 14 (87.50) |  |  | |
| Laparoscopic | 12 (17.65) | 2 (10.00) |  |  | 0 (0.00) | 2 (12.50) |  |  | |
| cTNM stage, n (%) |  |  | 0.034 |  |  |  | 1.000 |  | |
| I | 5 (7.35) | 0 (0.00) |  |  | 0 (0.00) | 0 (0.00) |  |  | |
| II | 18 (26.47) | 1 (5.00) |  |  | 1 (6.25) | 1 (6.25) |  |  | |
| III | 45 (66.18) | 19 (95.00) |  |  | 15 (93.75) | 15 (93.75) |  |  | |
| **BMI**: Body Mass Index; **MGC**: mucinous gastric adenocarcinoma; **PSM**: Propensity Score Matching | | | | | | | | |  |

**Supplement Table 4** Comparison of clinicopathological characteristics before and after PSM of mixed MGC patients

| Patient Characteristics | Before PSM | | |  | After PSM | | |  | |
| --- | --- | --- | --- | --- | --- | --- | --- | --- | --- |
|  | Surgical  (n=390) | Neoadjuvant  (n=68) | *P* value |  | Surgical  (n=103) | Neoadjuvant (n=56) | *P* value |  | |
| Age, Mean ± SD | 64.34 ± 8.98 | 64.20 ± 8.25 | 0.922 |  | 66.56 ± 9.52 | 64.20 ± 8.25 | 0.213 |  | |
| BMI, Mean ± SD | 22.36 ± 3.17 | 21.77 ± 2.58 | 0.230 |  | 21.64 ± 3.52 | 21.77 ± 2.58 | 0.844 |  | |
| Sex, n (%) |  |  | 0.157 |  |  |  | 0.649 |  | |
| Female | 85 (23.68) | 15 (33.33) |  |  | 13 (28.89) | 15 (33.33) |  |  | |
| Male | 274 (76.32) | 30 (66.67) |  |  | 32 (71.11) | 30 (66.67) |  |  | |
| Family history, n (%) |  |  | 0.456 |  |  |  | 0.634 |  | |
| No | 252 (70.19) | 34 (75.56) |  |  | 32 (71.11) | 34 (75.56) |  |  | |
| Yes | 107 (29.81) | 11 (24.44) |  |  | 13 (28.89) | 11 (24.44) |  |  | |
| Smoking history, n (%) |  |  | 0.087 |  |  |  | 0.384 |  | |
| No | 191 (53.20) | 30 (66.67) |  |  | 26 (57.78) | 30 (66.67) |  |  | |
| Yes | 168 (46.80) | 15 (33.33) |  |  | 19 (42.22) | 15 (33.33) |  |  | |
| Alcohol consumption, n (%) |  |  | 0.890 |  |  |  | 0.660 |  | |
| No | 243 (67.69) | 30 (66.67) |  |  | 28 (62.22) | 30 (66.67) |  |  | |
| Yes | 116 (32.31) | 15 (33.33) |  |  | 17 (37.78) | 15 (33.33) |  |  | |
| Adjuvant chemotherapy, n (%) |  |  | 0.002 |  |  |  | 1.000 |  | |
| No | 140 (39.00) | 7 (15.56) |  |  | 7 (15.56) | 7 (15.56) |  |  | |
| Yes | 219 (61.00) | 38 (84.44) |  |  | 38 (84.44) | 38 (84.44) |  |  | |
| Tumor size, n (%) |  |  | 0.561 |  |  |  | 0.525 |  | |
| ＜5cm | 192 (53.48) | 22 (48.89) |  |  | 19 (42.22) | 22 (48.89) |  |  | |
| ≥5cm | 167 (46.52) | 23 (51.11) |  |  | 26 (57.78) | 23 (51.11) |  |  | |
| Lymphovascular invasion, n (%) |  |  | 0.927 |  |  |  | 0.384 |  | |
| No | 149 (41.50) | 19 (42.22) |  |  | 15 (33.33) | 19 (42.22) |  |  | |
| Yes | 210 (58.50) | 26 (57.78) |  |  | 30 (66.67) | 26 (57.78) |  |  | |
| Perineural invasion, n (%) |  |  | 0.253 |  |  |  | 0.667 |  | |
| No | 184 (51.25) | 19 (42.22) |  |  | 17 (37.78) | 19 (42.22) |  |  | |
| Yes | 175 (48.75) | 26 (57.78) |  |  | 28 (62.22) | 26 (57.78) |  |  | |
| Tumor location, n (%) |  |  | 0.600 |  |  |  | 0.721 |  | |
| Upper third | 85 (23.68) | 14 (31.11) |  |  | 15 (33.33) | 14 (31.11) |  |  | |
| Middle third | 72 (20.06) | 10 (22.22) |  |  | 7 (15.56) | 10 (22.22) |  |  | |
| Lower third | 198 (55.15) | 21 (46.67) |  |  | 23 (51.11) | 21 (46.67) |  |  | |
| Entire | 4 (1.11) | 0 (0.00) |  |  | 0 (0.00) | 0 (0.00) |  |  | |
| Differentiation, n (%) |  |  | 0.424 |  |  |  | 0.796 |  | |
| Low | 273 (76.04) | 35 (77.78) |  |  | 36 (80.00) | 35 (77.78) |  |  | |
| Moderate | 73 (20.33) | 10 (22.22) |  |  | 9 (20.00) | 10 (22.22) |  |  | |
| Well | 13 (3.62) | 0 (0.00) |  |  | 0 (0.00) | 0 (0.00) |  |  | |
| Surgical procedure, n (%) |  |  | 0.004 |  |  |  | 0.431 |  | |
| Open | 244 (67.97) | 40 (88.89) |  |  | 43 (95.56) | 40 (88.89) |  |  | |
| Laparoscopic | 115 (32.03) | 5 (11.11) |  |  | 2 (4.44) | 5 (11.11) |  |  | |
| cTNM stage, n (%) |  |  | 0.002 |  |  |  | 0.482 |  | |
| I | 62 (17.27) | 0 (0.00) |  |  | 0 (0.00) | 0 (0.00) |  |  | |
| II | 73 (20.33) | 6 (13.33) |  |  | 3 (6.67) | 6 (13.33) |  |  | |
| III | 224 (62.40) | 39 (86.67) |  |  | 42 (93.33) | 39 (86.67) |  |  | |
| **BMI**: Body Mass Index; **MGC**: mucinous gastric adenocarcinoma; **PSM**: Propensity Score Matching | | | | | | | | |  |
